# Genomic Landscape and Phenotypic Assessment of *Cronobacter sakazakii* Isolated From Raw Material, Environment, and Production Facilities in Powdered Infant Formula Factories in China

**DOI:** 10.3389/fmicb.2021.686189

**Published:** 2021-07-20

**Authors:** Xin Gan, Menghan Li, Shaofei Yan, Xiaofei Wang, Wei Wang, Fengqin Li

**Affiliations:** Key Laboratory of Food Safety Risk Assessment, National Health Commission, China National Center for Food Safety Risk Assessment, Beijing, China

**Keywords:** *Cronobacter sakazakii*, powdered infant formula, antimicrobial susceptibility, biofilm, virulence genes, whole genome sequencing

## Abstract

*Cronobacter* is a foodborne pathogen associated with severe infections and high mortality in neonates. The bacterium may also cause gastroenteritis, septicemia, and urinary tract and wound infectious in adults. A total of 15 *Cronobacter* isolates collected from 617 raw materials and environment samples from Powdered Infant Formula manufacturing factories during 2016 in Shaanxi, China, were analyzed for antimicrobial susceptibilities, species identification, biofilm formation, and whole-genome sequencing. The results showed that all 15 isolates were *Cronobacter sakazakii*, while the antimicrobial susceptibility test showed that all 15 *C. sakazakii* were pan susceptible. Most isolates were able to produce a weak biofilm, and two isolates from soil samples produced a strong biofilm formation. All isolates were classified into seven STs including ST4, ST40, ST64, ST93, ST148, ST256, and ST494, with ST64 (4/15, 26.7%) being dominant, and most were clinically related. The isolates harbored at least 11 virulence genes and two plasmids, with one isolate being positive for all virulence genes. Phylogenetic and ANI analysis showed strong clustering by sequence types and isolates from different sources or regions with a similar genomic background. The fact that isolates were obtained from raw materials and environment samples of PIF facilities shared a close phylogeny with one another suggests that cross-contamination events may have occurred between the processing room and external environments, which may give rise to a recurring risk of a continuous contamination during production.

## Introduction

*Cronobacter* species are Gram-negative opportunistic pathogens that were originally referred to as yellow-pigmented *Enterobacter cloacae* according to its characteristics of yellow-pigment production and biochemical reactions (Urmenyi and White-Franklin, 1961). When subsequently reclassified, they were renamed as *Enterobacter sakazakii* following data from DNA hybridization studies, biochemical reactions, and antibiotic sensitivity tests. Later again, they were further reclassified by Iversen in 2008 based on 16S rRNA gene sequencing, ribotyping, DNA–DNA hybridization, and fluorescence labeled-amplified fragment length polymorphism fingerprinting (Iversen et al., 2008). *Cronobacter* species are considered to be a category A pathogen of relevance to powdered infant formula (PIF) as defined by FAO/WHO in 2004. It is known to cause severe infantile septicemia, necrotizing enterocolitis, meningitis, and serious neurological sequelae predominantly in neonates and low-birth-weight infants, especially infants who are premature or immune deficient, and the overall case fatality of invasive neonatal *Cronobacter* infection in Friedemann’s research was 26.9%; however, according to different clinical symptoms and regions, the mortality rate could rise to 100% (Friedemann, 2009). *Cronobacter* species have been isolated from infant food, beverages, processed food, plants, fresh produce, animal products, environments (e.g., dust, soil, and water), and households (Ueda, 2017). Neonates are infected through the consumption of PIF, or through contaminated bottles and utensils used for PIF preparation. Since the first *Cronobacter* clinical infection reported in UK, approximately 150 of *Cronobacter* infections in neonates with 26 deaths have been reported around the world (Iversen et al., 2008; Jaradat et al., 2014; Farmer, 2015). In 2002, the International Commission on Microbiological Specifications for Food classified *Cronobacter* as “severe risk for a restricted population, representing a threat of death or chronic sequelae” (ICMSF (International Commission on Microbiological Specifications for Foods), 2002).

*Cronobacter* adhere to both human intestinal epithelial and brain microvascular endothelial cell surfaces, which may be considered as the first step before colonization and infection (Townsend et al., 2007, 2008). After invasion of the intestinal cell, intracellular proliferation occurs; the bacteria then translocate to the apical side of the epithelia cell where they get released into the lamina propria and are able to spread systemically by way of lymphoid cells and macrophages. Once at extra-intestinal sites like the blood brain barrier, they then cause meningitis by currently unknown factors. Although multilocus sequence typing (MLST) is not used to evaluate the virulence of bacteria, lots of data have indicated that certain sequence types (STs) are more often associated with pathogenicity (Forsythe et al., 2014). *Cronobacter sakazakii* was a primary *Cronobacter* spp. causing clinical infections in neonates and adults (Forsythe, 2018). Accordingly, *C. sakazakii* ST4 is the predominant ST causing neonatal meningitis in comparison with ST1, ST8, ST12, ST21, ST64, and ST201 (Fei et al., 2017). Moreover, ST4 isolates show a stronger capacity of desiccation resistance, which may directly lead to the fact that ST4 is the predominantly found in PIF, suggesting a potential risk to infants (Fei et al., 2017).

Whole-genome sequencing (WGS) has been widely used as a powerful tool for characterization of *Cronobacter*, capable of reporting on virulence-related genes such as outer membrane proteins, efflux systems, iron acquisition systems, and hemolysins (Franco et al., 2011a,b; Joseph et al., 2013; Singh et al., 2015; Gao et al., 2017; Kim et al., 2017; Ye et al., 2017). In this study, a total of 15 *Cronobacter* isolates that came from 617 samples which consisted of PIF, raw materials, and environmental samples of two PIF manufacturing facilities in 2016 in Shaanxi, China, were sequenced and analyzed to investigate their associated genotypic and phenotypic virulence and persistence mechanisms.

## Materials and Methods

### Identification of *Cronobacter* From PIF Factories

**FIGURE 1:**
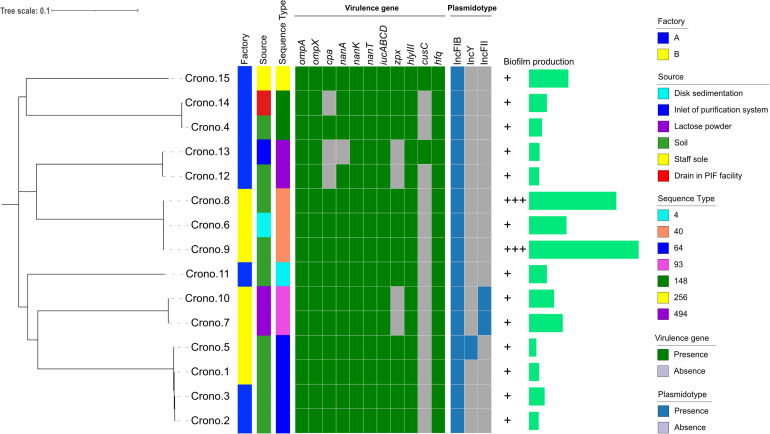
Phylogenetic tree and heat-map summary of presence of virulence genes and plasmids possessed by the 15 *C. sakazakii* isolates. The phylogenetic tree shown on the left was generated using CFSAN_SNP pipeline and FastTree v2.1 taking the core genome (3,440 core genes were analyzed) SNPs as input and was visualized with iTOL. Isolation information including PIF manufacturing facility location and sources as well as the sequence types are shown with stripes in different colors. The presence and absence of virulence genes and plasmidotype are shown with heat map. The biofilm production of all 15 isolates are shown with symbols (+ ∼ + +) and simple bar chart exhibiting their capacity of biofilm formation.

*Cronobacter* strains analyzed in this study were collected from a project (Gan et al., 2021) and stored in our laboratory, as shown in Table 1 and Figure 1. These strains were obtained from 617 samples in 2016 involving a processing room and items of two PIF factories in Shaanxi, China, including samples of PIF raw material ingredient, production facility environmental surfaces, PIF, and shoe soles of staff. The raw ingredients included lactose, whey powder, concentrated whey protein, galacto-oligosaccharide, fructo-oligosaccharides, DHA powder, ARA powder, casein calcium phosphate, α-lactalbumin, lactoferrin, bifidobacterium, and vegetable oil that were sampled in package directly. All isolates were identified by both VITEK 2 compact Gram-negative identification card analysis (bioMérieux, Marcy-l’Étoile, France) and amplification of the internal transcribed spacer (ITS) (ITS-F 5′-GGGTTGTCTGCGAAAGCGAA-3′, ITS-R 5′-GTCTTCGTGCTGCGAGTTTG-3′) by polymerase chain reaction (PCR) (Liu et al., 2006). Species identification of *Cronobacter* was carried out based on the RNA polymerase beta subunit (*rpoB*) gene according to previous studies (Stoop et al., 2009; Lehner et al., 2012). Reference strains including *C. sakazakii* (ATCC^TM^ 29544), *C. malonaticus* (DSM 18702), *C. turicensis* (DSM 18703), *C. dublinensis* subsp. *dublinensis* (DSM 18705), *C. dublinensis* subsp. *lausannensis* (DSM 18706), *C. dublinensis* subsp. *lactaridi* (DSM 18706), *C. universalis* (NCTC 9529), *C. muytjensii* (ATCC^TM^ 51329), and a *C. condimenti* isolate from University College Dublin were included as positive controls for PCR assays, while *E. coli* ATCC25922, *Enterobacter cloacae* CMCC45301, and *Salmonella* CMCC14028 were included as negative controls. Malonate utilization of all confirmed isolates was also tested by inoculating the fresh culture of each isolate in a malonate medium (Beijing Land Bridge Technology Ltd., Beijing, China) at 37°C for 24 h. All confirmed isolates were stored in Brain Heart Infusion 96 broth with 40% [v/v] glycerol (HopeBio, Qingdao, China) at −80°C.

**TABLE 1:** Information of 15 *C. sakazakii* isolates.

**ID**	**Accession number**	**AST**	**ST**	**Clonal complex**	**Genome size (bp)**	**G + C content (%)**	**Gene number**
Crono.1	JAGEMS000000000	S*	64	64	4,275,370	57.77	3980
Crono.2	JAGEZG000000000	S	64	64	4,283,664	56.34	3981
Crono.3	JAGEZH000000000	S	64	64	4,273,602	57.64	3980
Crono.4	JAGFJU000000000	S	148	16	4,476,549	57.15	4197
Crono.5	JAGFJV000000000	S	64	64	4,422,424	57.38	4161
Crono.6	JAGFJW000000000	S	40	40	4,502,936	57.30	4241
Crono.7	JAGFJX000000000	S	93	–	4,430,830	57.31	4113
Crono.8	JAGFJY000000000	S	40	40	4,503,421	57.22	4241
Crono.9	JAGFJZ000000000	S	40	40	4,500,991	57.32	4241
Crono.10	JAGFKA000000000	S	93	–	4,429,443	57.52	4108
Crono.11	JAGFKB000000000	S	4	4	4,444,221	57.29	4186
Crono.12	JAGFKC000000000	S	494	–	4,423,227	57.52	4072
Crono.13	JAGFKD000000000	S	494	–	4,475,585	57.51	4140
Crono.14	JAGFKE000000000	S	148	16	4,327,190	57.51	4081
Crono.15	JAGFKF000000000	S	256	–	4,515,171	57.47	4208

*^∗^S represented susceptible.*

### Antimicrobial Susceptibility Testing (AST)

All confirmed *Cronobacter* isolates were performed on AST using the Biofosun^®^ Gram-negative panels (Fosun Diagnostics, Shanghai, China) by broth dilution method (CLSI, 2019). The panel of antimicrobial compounds tested included tetracycline (TET), nalidixic acid (NAL), ciprofloxacin (CIP), sulfamethoxazole–trimethoprim (SXT), amoxicillin (AMX), ampicillin (AMP), ampicillin–sulbactam (SAM), gentamicin (GEN), cefotaxime (CTX), ceftazidime (CAZ), and imipenem (IMP). The *Escherichia coli* ATCC^TM^ 25922 strain was used as a quality control for the AST.

### Biofilm Production

Biofilm formation was assessed in a 96-well microtiter plate assay using minimal medium M9 (6 g/l Na_2_HPO_4_, 3 g/l KH_2_PO_4_, 0.5 g/l NaCl, 1 g/l NH_4_Cl, 2 mM MgSO_4_, 0.1% glucose, and 0.1 mM CaCl_2_) (Huang et al., 2015). After overnight growth at 37°C in tryptic soy broth medium (TSB; Beijing Land Bridge Technology Ltd., Beijing, China), 200 μl *Cronobacter* cell suspension was transferred into each microtiter well and incubated at 37°C for 72 h. After three brief washes with 200 μl phosphate-buffered saline (PBS) solution, and a 20-min fixation step with 200 μl methanol, all plates were stained with 200 μl 0.4% (w/v) crystal violet (CV) for 15 min and washed three times with 200 μl PBS for another 15 min. The stained biofilm was then dissolved with 200 μl 33% (v/v) acetic acid for 30 min. The biofilm formation was measured at 570 nm optical density (OD) in a microtiter plate reader (Tecan, Mannedorf, Switzerland). *Salmonella* Typhimurium ATCC^TM^ 14028, a strong biofilm-forming strain, was recruited as the positive control, and sterile minimal medium M9 was used as negative control for the biofilm production assays (Martins et al., 2013). These biofilm assays were performed in triplicate which included biological duplicates. On the basis of criteria described by Lee, OD at 570 nm (OD570) < 0.5 was defined as “weak,” OD570 ranging from 0.5 to 1.0 was “moderate,” and OD570 > 1 was “strong” (Lee et al., 2012).

### DNA Extraction and WGS

*Cronobacter* isolates were inoculated onto a tryptone soya agar (TSA) plate (Beijing Land Bridge Technology Ltd., Beijing, China) and incubated overnight at 37°C to obtain a pure culture. Genomic DNA (gDNA) was purified using a Tiangen TIANamp Bacteria DNA Kit (DP302-02, Tiangen Biotech Co., Ltd., China) according to the manufacturer’s procedures. The qualified gDNA (OD260/280 = 1.8–2.0 and concentration ≥20 ng/μl with a single DNA band) was then dispatched to Novogene for commercial sequencing. Illumina HiSeq protocols were carried out by Novogene (China) on an Illumina HiSeq platform. SOAP *de novo* v2.04 (Li et al., 2010), SPAdes, and AbySS were used for assembly, and CISA was used to integrate the assembly (Simpson et al., 2009; Bankevich et al., 2012; Lin and Liao, 2013).

### Genome Annotation and Bioinformatic Analysis

Prokka v1.12-beta (Seemann, 2014), KOALA v2.2^1^, and PubMLST^2^ were used to perform the genome annotation. Seven-gene MLST of *Cronobacter* was performed *in silico* for all the isolates studied by uploading the whole-genome sequence to the PubMLST *Cronobacter* spp. Database^3^. Virulence genes were selected based on a careful review of the literature (Franco et al., 2011a,b; Joseph et al., 2013; Singh et al., 2015; Gao et al., 2017; Kim et al., 2017; Ye et al., 2017) and then screened among the studied genomes using the BLASTN algorithm (Altschul et al., 1990) with minimum nucleotide identity and alignment length coverage of 90%. Plasmidotype was identified using the ABRicate^4^ software package, which contains the PlasmidFinder database (Carattoli et al., 2014). The antimicrobial resistance genes (AMR genes) were also identified using the ABRicate (see text footnote 4) software package, which contains the ResFinder, CARD, ARGannot, and NCBI databases (Carattoli et al., 2014).

### Phylogenetic Analysis

A maximum-likelihood phylogenetic tree was developed with the Center for Food Safety and Applied Nutrition and single-nucleotide polymorphism and single-nucleotide polymorphism (CFSAN_SNP) pipeline with FastTree v2.1 (Price et al., 2010; Burall et al., 2017). The phylogenetic tree was subsequently visualized through iTOL (Letunic and Bork, 2019). The average nucleotide identity (ANI) analysis was calculated using the Python module PYANI v0.1.3.2 for all *Cronobacter* genomes (Pritchard et al., 2015). In addition, to determine the underlying genomic population structure of the isolates, a core genome maximum likelihood phylogenetic tree clustered by different STs was also constructed with all available reference genomes downloaded from the PATRIC database (Wattam et al., 2017). In brief, all annotated genome assemblies were taken as input for pan genome analysis with core gene alignments through Roary (Page et al., 2015). Maximum likelihood phylogenies of the concatenated core gene alignments were constructed using FastTree (Price et al., 2010). The final tree was visualized with iTOL (Letunic and Bork, 2019).

### Nucleotide Sequence Accession Numbers

Accession numbers for draft sequence files (raw reads) are given in Table 1.

## Results

### Species Identification and AST of *Cronobacter*

A total of 15 *Cronobacter* strains were recovered from 617 samples including lactose powder, soil, and disk sedimentation (sampled by the TSA plates exposed in air of PIF manufacturing facilities for 15 to 20 min) and swabs taken from an air inlet, drain, and shoe soles of staff working in two PIF production facilities in Shaanxi, China, 2016 (Table 1 and Figure 1). All these isolates were identified as *C. sakazakii* using the *rpoB* PCR species identification method. Notably, five *C. sakazakii* isolates were found to be positive for malonate utilization (four were subsequently identified as ST64; the other one was ST148). AST results showed that all 15 *C. sakazakii* isolates were susceptible to all 11 tested antimicrobials (pan susceptible) (Table 1).

### Biofilm Formation

*Cronobacter sakazakii* were studied for biofilm formation using the microplate method with M9 minimal medium at 37°C, and the results showed that all 15 isolates could produce biofilm, although at different formation levels (Figure 1). Thirteen isolates (13/15, 86.7%) were able to produce a weak biofilm, and two strains collected from soil showed strong biofilm formation.

### MLST

The MLST results showed that seven STs were identified among 15 isolates, with ST64 (4/15, 26.7%) being the predominant ST that isolated from soil around the PIF facility, followed by three ST40 (3/15, 20.0%) with two from soil and one from disk sedimentation of the PIF processing room, two ST93 (2/15, 13.3%) collected from lactose powder, two ST148 (2/15, 13.3%) from soil and processing room drain, two ST494 (2/15, 13.3%) from soil and air inlet, a single isolate of ST256 (1/15, 6.7%) from staff members shoes’ soles, and one ST4 (1/15, 6.7%) from soil (Table 1 and Figure 1). The recognized clonal complexes for STs are also listed in Table 1.

### Genomic Sequence Information

A summary of the genome statistics for the 15 *C. sakazakii* isolates is provided in Table 1. The average total genome length of the *de novo* assembly of genomes was 4,419 kb with a range of 4,273 to 4,515 kb. The average total number of genes was 4,129 with a gene number range of 3,980 to 4,241 observed among the genomes. The average G + C content of these genomes was 57.4% with a range from 56.3 to 57.8%.

### Virulence Factors and AMR Genes Annotated in the Bacterial Genome and Plasmid Replicon Type

Nine types of genes related to virulence and toxin production were detected in this study. These include genes encoding for outer membrane proteins (*ompA*, *ompX*), plasminogen activator (*cpa*), sialic acid utilization (*nanAKT*), iron acquisition system (*iucABCD*), zinc-containing metalloprotease (*zpx*), hemolysin III (*hly*III), hemolysin expression-modulating protein (*hha*), *ibeB*-homologous (*cusC*), and RNA-binding protein (*hfq*). The details are shown in Figure 1 and Tables 2, 3. All 15 *Cronobacter* isolates carried the *ompA*, *ompX*, *nanKT*, *iucABCD*, *hly*III, *hha*, and *hfq* genes. Totally, 93.3% (14/15) isolates carried the *nanA* gene, while the *cpa*, *zpx*, and *cusC* genes were found in 80.0% (12/15), 73.3% (11/15), and 13.3% (2/15) isolates, respectively. A total of 14 (14/15, 93.33%) isolates were positive for all the *nanAKT* genes. Of note, one isolate (1/15, 6.7%) was found to harbor all of the 15 virulence genes. In all, six virulence gene patterns were found among 15 *C. sakazakii* isolates (Table 3). The AMR genes were also analyzed among all 15 *C. sakazakii* genomes. However, no AMR genes were identified by ABRicate software package against the ResFinder, CARD, ARGannot, and NCBI databases.

**TABLE 2:** Virulence genes harbored by *C. sakazakii* isolates.

**ID**	***ompA***	***ompX***	***cpa***	***nanA***	***nanK***	***nanT***	***iucABCD***	***zpx***	***hly III***	***hha***	***cusC***	***hfq***
Crono.1	+	+	+	+	+	+	+	+	+	+	-	+
Crono.2	+	+	+	+	+	+	+	+	+	+	-	+
Crono.3	+	+	+	+	+	+	+	+	+	+	-	+
Crono.4	+	+	+	+	+	+	+	+	+	+	-	+
Crono.5	+	+	+	+	+	+	+	+	+	+	-	+
Crono.6	+	+	+	+	+	+	+	+	+	+	-	+
Crono.7	+	+	+	+	+	+	+	-	+	+	-	+
Crono.8	+	+	+	+	+	+	+	+	+	+	-	+
Crono.9	+	+	+	+	+	+	+	+	+	+	-	+
Crono.10	+	+	+	+	+	+	+	-	+	+	-	+
Crono.11	+	+	+	+	+	+	+	+	+	+	-	+
Crono.12	+	+	-	+	+	+	+	-	+	+	-	+
Crono.13	+	+	-	-	+	+	+	-	+	+	+	+
Crono.14	+	+	-	+	+	+	+	+	+	+	-	+
Crono.15	+	+	+	+	+	+	+	+	+	+	+	+
Prevalence,%	100.0	100.0	80.0	93.3	100.0	100.0	100.0	73.3	100	100	13.3	100

**TABLE 3:** Patterns of virulence genes distribution in *C. sakazakii* isolates.

**Gene patterns**	***ompA***	***ompX***	***cpa***	***nanA***	***nanK***	***nanT***	***iucABCD***	***zpx***	***hly III***	***hha***	***cusC***	***hfq***	**Prevalence %**
I	+	+	+	+	+	+	+	+	+	+	-	+	60.0% (9/15)
II	+	+	+	+	+	+	+	-	+	+	-	+	13.3% (2/15)
III	+	+	+	+	+	+	+	+	+	+	+	+	6.7% (1/15)
IV	+	+	-	+	+	+	+	-	+	+	-	+	6.7% (1/15)
V	+	+	-	-	+	+	+	-	+	+	+	+	6.7% (1/15)
VI	+	+	-	+	+	+	+	+	+	+	-	+	6.7% (1/15)

Fifteen *C. sakazakii* isolates carried three types of plasmids, and all strains harbored the IncFIB plasmid. Besides, two isolates (2/15, 13.3%) carried the IncFII plasmid, and one isolate (1/15, 6.7%) carried the IncY plasmid.

### Whole-Genome Phylogenetic Characteristic of All *C. sakazakii* Isolates

According to the phylogenetic tree, 15 *C. sakazakii* showed strong clustering by their STs (Figure 1). Four ST64 isolates collected from two factories, carrying the same virulence gene pattern I, showed a close relationship and also had similar biofilm formation capacity; three of these strains had one pESA3 plasmid and the rest one carried two plasmids (Figure 1 and Table 3). Three ST40 isolates, harboring the same virulence gene pattern I and plasmidotype IncFIB, also showed a close relationship. Two of these ST40 isolates from soil had a strong biofilm formation capacity, while the other one from disk sedimentation of the processing room showed a weak capacity (Figure 1 and Table 3).

**FIGURE 2:**
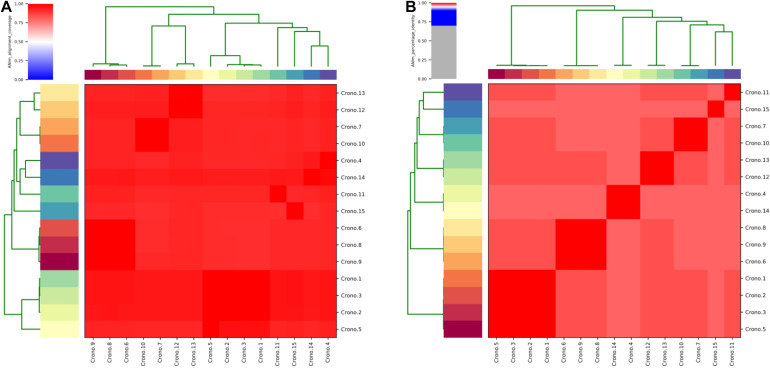
Average nucleotide identity (ANI) analysis of all 15 *C. sakazakii* genomes. **(A)** Heat map of the ANIm coverage for the studied genomes. The 15 genomes assignments as indicated at the source are given as row and column labels. Cells in the heat map corresponding to 50% coverage or greater are marked in red. Blue cells correspond to coverage of 50% or less. Color intensity fades as the comparisons approach 50% coverage. Color bars above and on the left of the heat map correspond to isolate ID for each genome in the analysis. Hierarchical clustering of the data in two dimensions is represented by dendrograms, constructed by simple linkage of ANIm percentage identities. This analysis identifies a single with a minimum aligned genome length above 50%. **(B)** ANIm percentage identity for the studied genomes. The 15 genomes as indicated at the source are given as row and column labels. Cells in the heat map corresponding to 95% ANIm sequence identity are colored red. Color intensity fades as the comparisons approach 95% ANIm sequence identity. Color bars above and to the left of the heat map correspond to isolate ID for each genome in the analysis. Hierarchical clustering of the analysis results in two dimensions is represented by dendrograms, constructed by simple linkage of ANIm percentage identities. The analysis indicates a single-level clade along the heat-map diagonal at the 95% identity threshold, suggesting that the current 15 studied *Cronobacter* genomes have a high sequence similarity at the nucleotide level.

The ANI analysis showed that 15 isolates could be divided into two clades: four *C. sakazakii* ST64 isolates from soil collected from two factories shared >99% of their total genome length with each other; 11 genomes shared >90% of their total genome length in their own clade and shared 92–96% when compared with those former four genomes (Figure 2). The alignment identity plot showed that they shared nucleotides of all genomes which had >97% similarity to each other (Figure 2), and isolates within the same ST which had a high similarity as shown by the four ST64 isolates from soil samples with >99% similarity and three ST40 isolates from disk and soil samples that also had >99% similarity.

**FIGURE 3:**
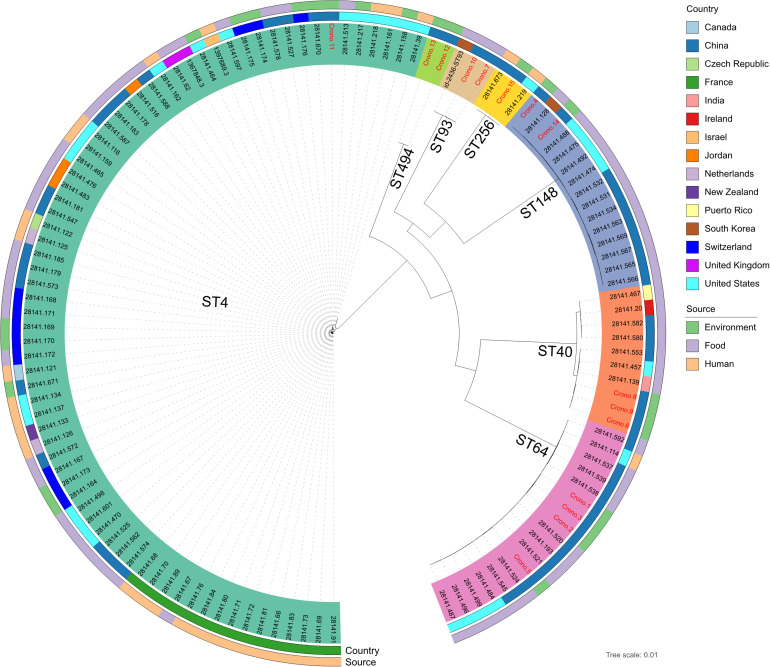
Whole-genome maximum likelihood phylogenetic trees of all 15 *C. sakazakii* genomes in this study combined with 108 reference genomes shared the same sequence types from PATRIC. Isolates that were clustered with the same color-coded label background shared the same sequence type, as marked on each clade. The ID font of genomes in this study and reference genomes were distinguished in red and black, respectively. The isolation information of countries and sources were also color-coded in the following rings.

A population structure analysis was reconstructed using a core-genome tree including all genomes of 15 studied *C. sakazakii* and an additional 108 available reference genomes from PATRIC that shared the same STs identified in this study (Figure 3 and Supplementary Table 1). The phylogenetic tree showed strong clustering by STs. Besides, no geographical or source specificities were found. *C. sakazakii* genomes in this study were clustered within reference Chinese genomes; for example, the Chinese mushroom isolate (28141.193) and other food isolates were clustered and close to our ST64 isolates but also had similarities with genomes from other countries. Similar observations were also found, showing that isolates from various sources were clustered with each other. Most environmental *C. sakazakii* in this study were found to be mixed with food and human related isolates.

## Discussion

*Cronobacter* contamination in PIF and infant foods could cause severe infections in neonates, particularly in low-birth-weight neonates, and has drawn great attention among international organizations and countries (Patrick et al., 2014). In our previous study, the contamination rate of *Cronobacter* in 119 PIF samples collected in China was 3.4% (Gan et al., 2018). This contrasts with a very early study that reported 14.2% of samples being positive for *Cronobacter* among 141 PIF samples studied (Muytjens et al., 1988). The detection of *Cronobacter* from PIFs has posed potential risk of infections in neonates and low-birth-weight infants, especially infants who are premature or immunodeficient. Following an earlier FAO-WHO risk assessment, this bacterium was categorized as a Category A bacterial pathogen associated with PIF with clear evidence of causality (Food and Agriculture Organization-World Health Organization (FAO-WHO), 2004). Therefore, the control of *Cronobacter* in PIF and its production environment is important in limiting the risk of neonatal infection through contaminated products. Strategies to prevent persistence in the PIF factory, including limiting the contamination on surfaces of processing facilities and surviving in raw material, are required. Thus, understanding the epidemiology of this bacterium in this context is a key step, toward development of targeted food safety controls.

In this study, 15 *Cronobacter* isolates cultured from raw materials, production facilities, and environment of two PIF factories were investigated by conventional bacteriological methods and WGS. All isolates were identified as *C. sakazakii*, being similar to those reported by Huang et al. (2015). In their study, 76 of 1,012 samples collected from 14 manufacturing factories in China during 2010 were *Cronobacter* positive, and 52 (52/76, 68.4%) were identified as *C. sakazakii* (Huang et al., 2015). The detection of *C. sakazakii* from different PIF production sources indicated that this bacterium can disseminate through raw ingredients, along with soil cross-contamination in the PIF production site. Our data suggested that PIF manufacturing factories should improve supervision of the environment around the factory and strengthen hygiene management in regard to raw materials. In this study, susceptibility testing of *C. sakazakii* showed pan susceptibility and lack of any AMR known genotypes. Our previous study showed that the antibiotic resistant rate was 1.92% (8/417) (Gan et al., 2015). However, in the study of Peng, 56 *C. sakazakii* were sensitive to SAM, CTX, CIP, meropenem, TET, piperacillin–tazobactam, and trimethoprim–sulfamethoxazole; 5 (8.9%) were resistant to chloramphenicol; 2 (3.6%) were resistant to GEN; and 31 (55.4%) were resistant to cephalothin (Fei et al., 2017). Further, *Cronobacter* cultured from food and clinical stool samples showed a 16% multidrug resistance in 90 isolates including two isolates that were resistant to more than five antibiotics along with six isolates that were resistant to six antibiotics (Li et al., 2020).

According to a study by Fei, ST4, ST1, and ST64 were the predominant STs among 70 *Cronobacter* isolated from PIF (Fei et al., 2015), and in the work of Lepuschitz, ST4, ST17, ST1, and ST40 were the dominant STs among their clinical isolates (Lepuschitz et al., 2019). Similar data were also found in our study showing that ST64 was the predominant ST (26.7%) followed by ST40 (20%). One ST4 (6.7%) isolate from a soil sample taken around the PIF factory was identified in our study, which was considered as the major ST associated with neonatal meningitis and has been proved to show a stronger desiccation-resistant capability than other STs (Joseph and Forsythe, 2011). Recently, ST494 and ST256 STs have been reported to be detected from neonatal *Cronobacter* meningitis infection cases in Brazil and China (Zeng et al., 2018; Costa et al., 2020). Furthermore, ST148 was also a dominant type with no reports associated with human infection. However, according to the PubMLST database, one ST148 isolate was obtained from a 64-year-old person’s blood sample in Denmark in 2009 (Li et al., 2019). The frequent detection of clinically relevant STs (four out of seven STs) highlights that it is necessary to investigate the distribution of these STs in PIF in China. Previous studies reported that some *C. sakazakii* had the capacity for malonate utilization (Iversen et al., 2008; Gopinath et al., 2018). In this study, five *C. sakazakii* isolates (four ST64, one ST148) could also utilize malonate, representing 33.3% (5/15) of all isolates, a higher number than that in an earlier study by Iversen (<5%). The malonate utilization is now considered as one typical biochemical reaction of *C. malonaticus* identification. Therefore, the observation of malonate utilization by *C. sakazakii* in this study indicated that the current identification methodology of *C. malonaticus* is inappropriate and may need to be improved.

Virulence genes were also analyzed in this study. The *ompA* and *ompX* genes in *C. sakazakii* play a critical role in both adherence and invasion through receptors which are located in both basolateral and apical host cell membrane surfaces (Kim et al., 2015). Iron is considered as an essential microelement for bacteria and also an important factor related to pathogenesis. Accordingly, the *cpa* and *iucABCD* genes are reported to be carried by virulence plasmids (Franco et al., 2011a,b). These virulence genes were also identified in genomes of *C. sakazakii* in our study. The *iucABCD* cluster participated in encoding a siderophore-mediated iron acquisition system to support bacterial growth in human hosts (Franco et al., 2011a). The plasmid-borne outer membrane protease Cpa has the ability to render serum resistance by cleaving complement components and efficient invasion by activating plasminogen and inactivating the plasmin inhibitor α2-AP (Franco et al., 2011b). Type III hemolysin and hemolysin expression-modulating proteins are potential virulence-related factors of *Cronobacter*, which have been reported in a recent study (Ye et al., 2017). The Hfq protein first discovered in *E. coli* plays an important role in the pathogenesis of various bacteria, as Hfq is regarded as a post-transcriptional global regulator and participates in the biogenesis of outer membrane proteins which could increase virulence *in vivo*, enhancing host cell adhesion and invasion *in vitro* (Kim et al., 2015). Considering that *C. sakazakii* is detected in the majority of clinical isolates in all age groups, the presence of the above virulence genes in all 15 isolates could contribute to risk of infection in vulnerable humans. Meanwhile, 93.3% (14/15) isolates carried the *nanAKT* genes that encode for sialic acid utilization and the uptake of sialic acid into bacteria, which are related to a number of virulence factors and could aid the bacterium to overcome the immune responses of the host (Singh et al., 2015). Furthermore, 12 isolates (80%) and 11 (73.3%) isolates carried the *cpa* gene and *zpx* gene, respectively. The *zpx* gene codes a zinc-containing metalloprotease and is considered as a putative virulence factor. It has been shown that Zpx can cause “rounding” for Chinese hamster ovary cells and has an ability to metabolize azocasein, azocoll, and insoluble casein, and the factor is also involved in helping the bacteria to cross the blood–brain barrier (Eshwar et al., 2018). Only two isolates (13.3%) including one ST256 and one ST494 carried the *cusC* gene. The ibeB homolog encoded by the *cusC* gene was reported to be associated with the invasion of human brain microvascular endothelial cells (Kim et al., 2017), and isolates harboring this gene may be one of the factors leading to neonatal meningitis similar to the two clinical cases reported in China and Brazil (Zeng et al., 2018; Costa et al., 2020). Of note, the ST256 *C. sakazakii* cultured from the shoe soles of staff working in PIF facility harbored all 15 virulence genes, suggesting that the virulence factors encoded by these genes might be responsible for severe pathogenicity exerted by this strain. Once it contaminated products and is consumed by infants, it could endanger consumers’ health and lead to serious infection symptoms.

In this study, the phylogenetic analysis of 15 *C. sakazakii* showed that isolates with the same STs exhibited a close genetic relationship and also carried similar virulence gene patterns. Four ST64 isolates were collected from two factories, and these showed a high similarity at a nucleotide level suggestive of a common ancestor. The phylogenetic relationship of three ST40 and two ST148 isolates also showed a high similarity and revealed that these isolates may evolve from the same ancestral species, respectively. Isolates with the same STs were obtained from both the interior and exterior environments of the PIF-processing site, suggesting that there may exist potential cross-contamination routes.

Population structure analysis including 15 genomes in this study and an additional 108 reference genomes further revealed that the STs of *C. sakazakii* formed their own distinct cluster, which was in agreement with previous studies (Lee and Andam, 2019; Jang et al., 2020). Therefore, it is essential to recognize more STs to aid in epidemiological investigations and risk assessments of this pathogen. Furthermore, *C. sakazakii* have been reported to be detected in imported PIF or dairy products in China and other countries (Guo et al., 2018; Valenci et al., 2019; Wang et al., 2019; Pakbin et al., 2020). Our data showed that the genome clusters had no geographical nor source specificities, which is in agreement with a recent research (Guo et al., 2018). The similar genomic background may be instrumental in the adaptive capability of those isolates, giving rise to a potential challenge for tracing of such pathogen from different regions or sources (Lee and Andam, 2019). However, more experimental evidence and more extensive sampling is needed to verify this.

## Conclusion

In conclusion, 15 *Cronobacter* isolates collected from raw material, environment, and production facilities of PIF manufacturing factories were identified with their AST phenotype, ST, and virulence genes. All *Cronobacter* in our study were identified as *C. sakazakii*, and ST64 was the predominant ST. All 15 *C. sakazakii* isolates harbored at least 12 virulence genes tested, and one ST256 isolate carried all 15 genes. Consumption of products contaminated by this bacterium will pose a potential threat to neonatal health. Isolates with the same STs were obtained from both inside and outside the environment of the processing room, indicating that there existed potential cross-contamination between both. Our observation shows that genomes of imported and domestic isolates had similar challenges for tracing *C. sakazakii* strains. The application of WGS could contribute to better understanding the pathogenicity of *C. sakazakii* and revealing the genomic relationship of isolates collected from different sources.

## Data Availability Statement

The datasets presented in this study can be found in online repositories. The names of the repository/repositories and accession number(s) can be found in the article/Supplementary Material.

## Author Contributions

XG contributed to the experimental studies and writing the manuscript. ML contributed to the WGS. SY contributed to the data analysis. XW contributed to the experimental studies. WW contributed to the WGS data analysis and manuscript editing. FL contributed to the study design and manuscript revision. All authors contributed to the article and approved the submitted version.

## Conflict of Interest

The authors declare that the research was conducted in the absence of any commercial or financial relationships that could be construed as a potential conflict of interest.

## Footnotes

1 https://www.kegg.jp/blastkoala/

2 https://pubmlst.org/organisms/cronobacter-spp

3 http://pubmlst.org/cronobacter/

4 https://github.com/tseemann/abricate
