# Supplementary material for: Genomic Landscape and Phenotypic Assessment of Cronobacter sakazakii Isolated From Raw Material, Environment, and Production Facilities in Powdered Infant Formula Factories in China
Source: Front Microbiol. 2021 Jul 20;12:686189. doi: 10.3389/fmicb.2021.686189 (PMC8329244; doi:10.3389/fmicb.2021.686189)
Supplement: Supplementary file 1 [file Table_1.docx]

**Supplementary Table S1: Genome metadata of the reference genomes obtained from the PATRIC database that used for phylogenetic analysis in this study.**

| **Genome ID** | **Assembly Accession** | **BioSample Accession** | **Isolation Source** | **Isolation Name** | **Isolation Country** | **Collection Date** | **MLST** |
| --- | --- | --- | --- | --- | --- | --- | --- |
| 28141.114 | GCF_001735605.1 | SAMN04329632 | bronchial wash | Human | United States | 1973 | ST64 |
| 28141.116 | GCF_001922925.1 | SAMN06100260 | alimentary canal | Human | United States | 2012 | ST4 |
| 28141.121 | GCF_001971115.1 | SAMN02780890 | Clinical | Human | Canada | 1990 | ST4 |
| 28141.122 | GCA_001971125.1 | SAMN02780899 | feces of 74 years old adult | Human | Czech Republic | 2003 | ST4 |
| 28141.125 | GCF_001971195.1 | SAMN02780907 | 1 day old infant | Human | Netherlands | 1977 | ST4 |
| 28141.126 | GCF_001971205.1 | SAMN02780981 | Clinical | Human | Netherlands | 1983 | ST4 |
| 28141.128 | GCA_001971255.1 | SAMN02796098 | Yoojeon food spices | Food | South Korea | 2005 | ST148 |
| 28141.133 | GCF_001971365.1 | SAMN02796166 | fecal isolate from infant with meningitis | Human | New Zealand | 2005 | ST4 |
| 28141.134 | GCF_001971375.1 | SAMN02796169 | CSF isolate from a 1 month old infant with meningitis | Human | United States | 2003 | ST4 |
| 28141.137 | GCF_001971445.1 | SAMN02796170 | CSF isolate of a 6 weeks old infant with non fatal brain abscess | Human | United States | 2003 | ST4 |
| 28141.139 | GCF_001972245.1 | SAMN02776191 | Ajwan cumin seeds | Food | India | 2005 | ST40 |
| 28141.158 | GCA_002075415.1 | SAMN06100269 | surface | Environment | United States | 2012 | ST4 |
| 28141.159 | GCA_002075425.1 | SAMN06100275 | alimentary canal | Human | United States | 2012 | ST4 |
| 28141.161 | GCA_002075445.1 | SAMN06100265 | alimentary canal | Human | United States | 2012 | ST4 |
| 28141.162 | GCA_002075495.1 | SAMN06100264 | alimentary canal | Human | United States | 2012 | ST4 |
| 28141.164 | GCA_002075535.1 | SAMN06124514 | PIF Production Facility Environment | Environment | Switzerland | 2011 | ST4 |
| 28141.167 | GCA_002075575.1 | SAMN06124515 | PIF Product | Food | Switzerland | 2012 | ST4 |
| 28141.168 | GCA_002075615.1 | SAMN06124517 | PIF Control | Food | Switzerland | 2011 | ST4 |
| 28141.169 | GCA_002075635.1 | SAMN06124501 | PIF Production Facility Environment | Environment | Switzerland | 2011 | ST4 |
| 28141.171 | GCA_002075655.1 | SAMN06124516 | PIF Control | Food | Switzerland | 2011 | ST4 |
| 28141.172 | GCA_002075695.1 | SAMN06124499 | PIF Control | Food | Switzerland | 2011 | ST4 |
| 28141.173 | GCA_002075715.1 | SAMN06124507 | PIF Production Facility Environment | Environment | Switzerland | 2012 | ST4 |
| 28141.174 | GCA_002075725.1 | SAMN06124508 | PIF Production Facility Environment | Environment | Switzerland | 2012 | ST4 |
| 28141.175 | GCA_002075735.1 | SAMN06124506 | PIF Production Facility Environment | Environment | Switzerland | 2012 | ST4 |
| 28141.176 | GCA_002075775.1 | SAMN06124513 | PIF Production Facility Environment | Environment | Switzerland | 2011 | ST4 |
| 28141.178 | GCA_002094435.1 | SAMN05412989 | deli meat samples | Food | China | 2015 | ST4 |
| 28141.179 | GCA_002094475.1 | SAMN05412997 | fried rice or noodles samples | Food | China | 2013 | ST4 |
| 28141.181 | GCA_002094495.1 | SAMN05412991 | cold noodles in sauce samples | Food | China | 2012 | ST4 |
| 28141.183 | GCA_002094555.1 | SAMN05412992 | deli meat samples | Food | China | 2012 | ST4 |
| 28141.185 | GCA_002094585.1 | SAMN05412993 | cold noodles in sauce samples | Food | China | 2012 | ST4 |
| 28141.193 | GCA_002094755.1 | SAMN05413005 | mushroom | Food | China | 2015 | ST64 |
| 28141.217 | GCA_002114505.1 | SAMN06100274 | surface | Environment | United States | 2012 | ST4 |
| 28141.218 | GCA_002114515.1 | SAMN06100273 | alimentary canal | Human | United States | 2012 | ST4 |
| 28141.219 | GCA_002114555.1 | SAMN06100272 | alimentary canal | Human | United States | 2012 | ST256 |
| 28141.39 | GCF_000974965.1 | SAMN03371494 | cerebrospinal fluid from a fatal case of infantile meningitis | Human | United States | 2008 | ST4 |
| 28141.457 | GCA_002942065.1 | SAMN08397391 | Food, Dried Chocolate Organic Shake | Food | United States | 2016 | ST40 |
| 28141.464 | GCA_002942205.1 | SAMN08397408 | Environmental, Dairy Plant | Environment | United States | 2005 | ST4 |
| 28141.465 | GCA_002942225.1 | SAMN08397394 | Food, Instant Oatmeal with Strawberries and Cream | Food | United States | 2005 | ST4 |
| 28141.467 | GCA_002942265.1 | SAMN08397392 | Food, Whole Grain, Corn | Food | Puerto Rico | 2013 | ST40 |
| 28141.474 | GCA_002942405.1 | SAMN08397390 | Food, Dried Cut Carrots | Food | United States | 2005 | ST148 |
| 28141.475 | GCA_002942415.1 | SAMN08397389 | Food, Dried Cut Carrots | Food | United States | 2005 | ST148 |
| 28141.476 | GCA_002974355.1 | SAMN08611295 | Chamomile | Food | Jordan | 2008 | ST4 |
| 28141.483 | GCA_002974495.1 | SAMN08611279 | Fennel | Food | Jordan | 2008 | ST4 |
| 28141.484 | GCA_002974505.1 | SAMN08611380 | Spices | Food | United States | 2015 | ST64 |
| 28141.487 | GCA_002974575.1 | SAMN08611376 | Spices | Food | United States | 2015 | ST64 |
| 28141.488 | GCA_002974595.1 | SAMN08611289 | Sodium Caseinate | Food | United States | 2005 | ST148 |
| 28141.492 | GCA_002974675.1 | SAMN08611365 | Sodium Caseinate | Food | United States | 2004 | ST148 |
| 28141.496 | GCA_002974755.1 | SAMN08611382 | Spices | Food | United States | 2015 | ST64 |
| 28141.498 | GCA_002974795.1 | SAMN08611379 | Organic Soy ISO III | Food | United States | 2004 | ST4 |
| 28141.499 | GCA_002974805.1 | SAMN08611378 | Spices | Food | United States | 2015 | ST64 |
| 28141.513 | GCA_002975095.1 | SAMN08611276 | Organic Soy | Food | United States | 2004 | ST4 |
| 28141.516 | GCA_002976495.1 | SAMN08611288 | Spices | Food | Jordan | 2008 | ST4 |
| 28141.521 | GCA_002976665.1 | SAMN07484014 | food | Food | China | 2011 | ST64 |
| 28141.524 | GCA_002976735.1 | SAMN07484009 | food | Food | China | 2012 | ST64 |
| 28141.525 | GCA_002976755.1 | SAMN07484012 | food | Food | China | 2011 | ST4 |
| 28141.527 | GCA_002976795.1 | SAMN07484008 | food | Food | China | 2011 | ST4 |
| 28141.531 | GCA_002976875.1 | SAMN07484004 | food | Food | China | 2012 | ST148 |
| 28141.532 | GCA_002976895.1 | SAMN07484003 | food | Food | China | 2012 | ST148 |
| 28141.534 | GCA_002976935.1 | SAMN07484001 | food | Food | China | 2012 | ST148 |
| 28141.537 | GCA_002976995.1 | SAMN07483998 | food | Food | China | 2016 | ST64 |
| 28141.538 | GCA_002977005.1 | SAMN07483997 | food | Food | China | 2016 | ST64 |
| 28141.539 | GCA_002977035.1 | SAMN07483996 | food | Food | China | 2016 | ST64 |
| 28141.545 | GCA_002977155.1 | SAMN07483990 | food | Food | China | 2011 | ST64 |
| 28141.547 | GCA_002977195.1 | SAMN07483988 | food | Food | China | 2012 | ST4 |
| 28141.553 | GCA_002977315.1 | SAMN07483982 | food | Food | China | 2012 | ST40 |
| 28141.562 | GCA_002977495.1 | SAMN07483972 | food | Food | China | 2007 | ST4 |
| 28141.563 | GCA_002977515.1 | SAMN07483969 | food | Food | China | 2007 | ST148 |
| 28141.565 | GCA_002977575.1 | SAMN07483970 | food | Food | China | 2007 | ST148 |
| 28141.566 | GCA_002977555.1 | SAMN07483971 | food | Food | China | 2007 | ST148 |
| 28141.567 | GCA_002977595.1 | SAMN07483968 | food | Food | China | 2007 | ST148 |
| 28141.569 | GCA_002977635.1 | SAMN07483966 | food | Food | China | 2007 | ST148 |
| 28141.572 | GCA_002977685.1 | SAMN07483963 | food | Food | China | 2007 | ST4 |
| 28141.573 | GCA_002977715.1 | SAMN07483962 | food | Food | China | 2007 | ST4 |
| 28141.574 | GCA_002977735.1 | SAMN07483961 | food | Food | China | 2007 | ST4 |
| 28141.578 | GCA_002977785.1 | SAMN07483957 | food | Food | China | 2007 | ST4 |
| 28141.582 | GCA_002977895.1 | SAMN07483954 | food | Food | China | 2007 | ST40 |
| 28141.587 | GCA_002977985.1 | SAMN07483947 | food | Food | China | 2015 | ST4 |
| 28141.588 | GCA_002977995.1 | SAMN07483949 | food | Food | China | 2012 | ST4 |
| 28141.592 | GCA_002978085.1 | SAMN07483942 | food | Food | China | 2015 | ST64 |
| 28141.597 | GCA_002993205.1 | SAMN08611274 | Unknown food powder | Food | United States | 2004 | ST4 |
| 28141.601 | GCA_003177155.1 | SAMN08611383 | Organic Soy Powder | Food | United States | 2004 | ST4 |
| 28141.62 | GCF_001277275.1 | SAMN03938714 | milk | Food | United Kingdom | 1980 | ST4 |
| 28141.66 | GCF_001308935.1 | SAMN02839386 | feces from infant with NECI | Human | France | 1994 | ST4 |
| 28141.67 | GCF_001308945.1 | SAMN02839371 | reconstituted formula which had not been fed to infant | Food | France | 1994 | ST4 |
| 28141.671 | GCA_003858325.1 | SAMN08427279 | Environment | Environment | China | 2016 | ST4 |
| 28141.673 | GCA_003955925.1 | SAMN08928172 | Brain abscess fluid | Human | China | 2015 | ST256 |
| 28141.68 | GCF_001308955.1 | SAMN02786796 | trachea | Human | France | 1994 | ST4 |
| 28141.69 | GCF_001308965.1 | SAMN02838910 | skin from infant with NECII | Human | France | 1994 | ST4 |
| 28141.71 | GCF_001309035.1 | SAMN02840767 | trachea from infant with septicemia | Human | France | 1994 | ST4 |
| 28141.72 | GCF_001309045.1 | SAMN02840765 | feces from infant with NECII | Human | France | 1994 | ST4 |
| 28141.73 | GCF_001309055.1 | SAMN02840768 | throat from infant with NECII | Human | France | 1994 | ST4 |
| 28141.76 | GCF_001309135.1 | SAMN02840782 | conjunctivae of infant with NECII | Human | France | 1994 | ST4 |
| 28141.81 | GCF_001309225.1 | SAMN02840809 | feces from infant with NECII | Human | France | 1994 | ST4 |
| 28141.83 | GCF_001309275.1 | SAMN02840819 | infant with NECI | Human | France | 1994 | ST4 |
| 28141.84 | GCF_001309295.1 | SAMN02840976 | feces from infant with NECII | Human | France | 1994 | ST4 |
| 28141.89 | GCF_001309415.1 | SAMN02837661 | feces from asymptomatic infant | Human | France | 1994 | ST4 |
| 28141.91 | GCF_001309435.1 | SAMN03650261 | peritoneal fluid from infant with NECIII | Human | France | 1994 | ST4 |
| 1367848.3 | GCA_000463095.1 | SAMN02261881 | milk powder | Food | United Kingdom | 1950 | ST4 |
| 1397689.3 | GCA_000467775.1 | SAMN02470723 | cerebral spinal fluid | Human | Israel | 2000 | ST4 |
| 28141.170 | GCA_002075645.1 | SAMN06124504 | PIF Production Facility Environment | Environment | Switzerland | 2012 | ST4 |
| 28141.20 | GCA_000698225.1 | SAMN02767826 | swab from food processing facility | Environment | Ireland | 2008 | ST40 |
| 28141.470 | GCA_002942325.1 | SAMN08397404 | Food, Organic Soy ISO | Food | United States | 2006 | ST4 |
| 28141.520 | GCA_002976645.1 | SAMN07484017 | food | Food | China | 2015 | ST64 |
| 28141.580 | GCA_002977855.1 | SAMN07483955 | food | Food | China | 2007 | ST40 |
| 28141.670 | GCA_003858315.1 | SAMN08427299 | Environment | Environment | China | 2016 | ST4 |
| 28141.70 | GCF_001309015.1 | SAMN02840766 | trachea from infant with NECII | Human | France | 1994 | ST4 |
| 28141.80 | GCF_001309215.1 | SAMN02840816 | sputum | Human | France | 1994 | ST4 |
| 28141.447 | GCA_002532375.1 | SAMN07205645 | Food ingredient | Food | South Korea | 2009 | ST93 |
